# Patterns of Nutrition and Dietary Supplements Use in Young Egyptian Athletes: A Community-Based Cross-Sectional Survey

**DOI:** 10.1371/journal.pone.0161252

**Published:** 2016-08-16

**Authors:** Safaa Tawfik, Nehal El Koofy, Eman Mohamed Ibraheim Moawad

**Affiliations:** 1 National Nutrition Institute, Ministry of Health, Cairo, Egypt; 2 Department of Pediatrics, Faculty of Medicine, Cairo University, Cairo, Egypt; Universitat de les Illes Balears, SPAIN

## Abstract

The aim of this study was to investigate the pattern of basic and sport nutrition as well as perspectives of young Egyptian athletes. Structured interview survey measuring knowledge, attitudes, beliefs and behaviors about basic and sport nutrition was administered to adolescent athletes recruited from 4 sport clubs and 2 fitness centers in Greater Cairo governorate. A total of 358 participants aged 13–18 years completed questionnaires. Basic nutrition knowledge was reasonable in almost all domains except fast food. Fixed breakfast (78.5%), home meals (lunch, 70.7%), and healthy snacks (55.8%) were the most positive features of the basic dietary pattern. More than 70% perceived themselves as knowledgeable about sport nutrition. The prevalence rate of sport supplement intake was (48.9%, n = 175), predominantly sport drinks (66.9%) and creatine (54.3%). Coaches were the primary source of sport nutrition information. Forty-four percent of participants (n = 77/175) reported supplement consumption during competition seasons only. Better physical appearance and enhancement of athletic performance were the major motivations for supplement intake. These findings indicate the necessity of a comprehensive nutrition education program targeting not only athletes and parents, but also coaching staff, health trainers and all sport team officials.

## Introduction

Good nutritional knowledge and adequate nutrient intake have been perceived as the key components that play a basic part in enhancing athletic performance [1]. In a joint position statement, the American College of Sports Medicine, American Dietetic Association, and Dietitians of Canada reported that “physical activity, athletic performance, and recovery from exercise are enhanced by optimal nutrition [2].”

Over the last 20 years, a growing number of young athletes have been participating at higher levels of competition [1]. As adolescents, the energy demands are increased and vary based on gender and level of physical activity [3]. However, several previous studies have demonstrated that increased energy requirements are not properly met in young athletes, especially during competition periods [3–5]. Most of them are unable to make appropriate nutritional choices due to poor dietary knowledge and education [6, 7].

The prevalence rates of supplement use among athletes were estimated to range from 32% to as high as 90% [8, 9]. Dietary supplements are defined as any products, apart from tobacco, expected to supplement the diet that contains one or more dietary ingredients [10]. Large number of children and adolescent athletes currently use at least one or more of sport supplements to boost their athletic performance [11, 12]. However, there are few studies on nutrition and sport supplement use in young athletes available from developing countries [13]. Adolescents from such communities receive much less attention and insufficient resources of nutrition information, with subsequent misjudgment of health-related choices [14].

Therefore, the present study aimed to investigate the pattern of dietary supplement use, supplement-related knowledge, beliefs, attitudes and practices among young Egyptian athletes in a variety of different sports. We also aimed to identify factors which influenced supplementation, perceived barriers for use, and sources of information regarding supplements among the study participants.

## Materials and Methods

A cross-sectional descriptive survey design was given to a convenience sample of young athletes gathered from four non-profit sport clubs [15] (6^th^ October, Al-Ahly, Heliopolis and Gifted Athletes School) and two fitness centers, representing different demographic regions in Greater Cairo governorate, between January to May 2015.

Eligible participants were 358 Egyptian adolescents aged 13–18 years, recruited from 11 different sports. To be included in the study, athletes should train ≥ 4 hours per week for the preceding 6 months [16]. The sample was made up of 76 competitive athletes (defined as an individual who participates in “competitive physical activities” or sports/games that require physical strength, agility, or stamina) [17] and 282 recreational athletes (defined as an individual who is physically active, but does not train for competition at the same level of intensity and focus as a competitive athlete) [17]. Thirty-seven competitive athletes were ranked at the district level, 34 were at the regional level, and 5 were at the national level.

Based on sport groups, participants were categorized into ball game, endurance, weight class, and anti-gravity sports [18]. The sampled population was chosen to ensure a range of team, individual as well as gender-specific sports. In terms of popularity, football is the most prevalent sport disseminating among the Egyptian community [19]; thus adequate number of participants were selected to ensure appropriate presentation of such sport.

Athletes with special needs practicing sports, local or traditional sports which are not widely practiced, and recreational activities e.g. walking were excluded from the survey.

The study was approved by Ethics Research Board of National Nutritional Institue, The General Organization Teaching Hospitals and Institute, Arab Republic of Egypt. Informed verbal consent was obtained from all parents or legal guardians before being enrolled in the study. Verbal assent was obtained from all respondents (≤18 years).

### Study instrument

The study was carried out by a trained team of five postgraduate students at "National Nutrition Institute" as a substantial part of the diploma project. The reliability of our study was improved through conducting training aimed to minimize the inter-observer variation in experience and to improve their efficacy in collecting data. A workshop was held to train the study investigators how to implement the survey activities in the practical field. A brief standard manual was provided to the field investigators that clarified how to present instructions to participants and answer questions about individual items.

Data were collected using a structured interview questionnaire adapted from previously validated work and published literature [20,21]. The English version of the revised questionnaire was translated into Arabic followed an established forward-backward translation procedure with respect to the recommendations of Sartorius and Kuyken [22].

A preliminary small-scale pilot study was conducted on 55 young athletes before executing the field survey. Based on the received feedback, some questions were modified to fit the study requirements. The new questionnaire was reviewed by three experts in the field of nutrition for content validity. Further proposed changes were made and the questionnaire was approved to serve as the data-gathering instrument.

The questionnaire consisted of 30 questions, divided into four main parts. Items of the first part collected the detailed demographic and personal information of the study participants such as age, gender, self-reported weight and height. The second part of the questionnaire was directed for a short assessment of dietary behavior during training and off-season periods. The third part was structured to measure athlete's knowledge of sports nutrition and sources of these nutrition information. The last part of the questionnaire has focused on a comprehensive analysis of knowledge, beliefs, attitudes and practice of the young athletes towards the dietary supplements.

To ensure the quality control on all phases of data collection, completed questionnaires were submitted and checked for missing information on a daily basis. Feedback was provided to research supervisor before the next day’s field study.

### Data analysis

Data were prepared in Microsoft Excel 2010 and analyzed with SPSS Version 16 (SPSS for Windows, Version 16.0. Chicago, SPSS Inc.). Data were statistically described in terms of mean ± standard deviation (± SD), median and range, or frequencies (number of cases) and percentages when appropriate. Questionnaires with missing values were excluded from the present analysis. The differences between proportions were assessed using the p-value for heterogeneity. Chi square and Fisher’s exact tests were used to estimate differences in qualitative variables. A p-value less than 0.05 was considered statistically significant.

## Results

Eligible participants included 358 adolescents aged 13–18 years old from 4 sport clubs and 2 fitness centers in Greater Cairo governorate. Participants demographic data are shown in Table 1. The mean age of respondents was 14.3 years (SD = 2.5) with significantly more males participating than females (56.4%, p < 0.001), giving a male to female ratio of 1.3:1. The years of practice ranged from 0.5 to 12 years with an average of 4.6 years. Based on self-perception of weight, 47.7% (n = 171) of participants described themselves as being in the normal weight range (20–25 kg/m²). The study young athletes spent an average of 4.8 hours weekly on training. Ball games (36.6%), predominantly football, were the most popular forms of sport among the study population (p < 0.001) Table 1.

**Table 1:** Characteristics of the study participants.

	Total respondents (n = 358)		*P* value
	Mean ± Standard deviation (range)	Number (%)	
**Gender**			
Males	-	202 (56.4)	**<0.001**
Females	-	156 (43.6)	
**Age (year)**	14.3 ± 2.5 (13–18)	-	
**Self-reported weight (Kg)**	63.6 ± 14.2 (35–100)	324 (90.5)	
**Self-reported height (cm)**	166.9 ± 11.1 (145–198)	311(86.9)	
**Body mass index (BMI) (Kg/m²)**	23.02 ± 3.3 (16.7–32.1)	289 (80.7)	
Lean (less than 20 kg/m2)	-	61(21.1)	**<0.001**
Normal weight (20–25 kg/m2)	-	171(59.2)	
Overweight (25–30 kg/m2) or obese (> 30 kg/m2)	-	57(19.7)	
**Sport category**			
**1. Ball games**		131(36.6)	**<0.001**
Football	-	83(23.2)	
Basketball	-	21(5.9)	
Volleyball		27(7.5)	
**2. Endurance**		129 (36)	
Swimming	-	77 (21.5)	
Distance running	-	45 (12.5)	
Cycling		7 (2)	
**3. Weight class**	-	104 (29)	
Wrestling	-	35 (9.8)	
Boxing	-	23 (6.4)	
Kickboxing	-	37 (10.3)	
Olympic weightlifting	-	9 (2.5)	
**4. Anti-gravity**		20 (5.6)	
**Practice durationᵃ (year)**	4.6 ± 2.6 (0.5–12)	-	
**Training/ week ᵃ (days)**	4.97 ± 1.1 (2–7)	-	
**Training/ week ᵃ (hours)**	4.8 ± 1.2 (4–10)	-	

Table 2 provides the results of responses to the basic dietary knowledge and behaviors of all the individuals studied. More than eighty-six percent of the study subjects (n = 312) had a significantly regular lunch intake compared to other meals (p = 0.005). The majority of them used to eat home-cooked meals including breakfast (n = 242, 67.6%), lunch (n = 253, 70.7%) and dinner (n = 188, 52.5%).

**Table 2:** Distribution of responses of young athletes to nutrition knowledge and behavior.

	Yes		*P* value
	Number (n)	%	
**What are your fixed meals?**			
Breakfast	281	78.5	**0.005**
Lunch	312	87.2	
Dinner	304	84.9	
**Do you have any snacks?**	337	94	
Once	113	33.5	0.625
Twice	114	33.8	
≥ three times	110	32.7	
**Where do you usually have snacks?**			
Home	89	26.4	**<0.001**
Outdoor	248	73.6	
**What are types of snacks?**			
Healthy	188	55.8	**0.004**
Unhealthy	149	44.2	
**Do you have fast food every week?**	313	87.4	
Once	181	57.8	**<0.001**
2–3 times	105	33.5	
> 3 times	27	8.6	
**Do you follow a special diet?**	209	58.4	
Training	67	32.1	**<0.001**
Competitions	49	23.4	
Ongoing	93	44.5	

Of those who reported to have routine daily snacks (n = 337), 55.8% had significantly consumed healthy snacks (p = 0.004), defined as any food item that was typically considered to be consumed between regular meals and provided the same food recommended for an athlete’s daily diet (e.g. Fruits, whole grains, lean protein).

As shown in Table 2, the percentage of adolescent athletes (n = 209) following a special diet increased significantly during their competition season versus out of season (p < 0.001).

**Fig 1:**
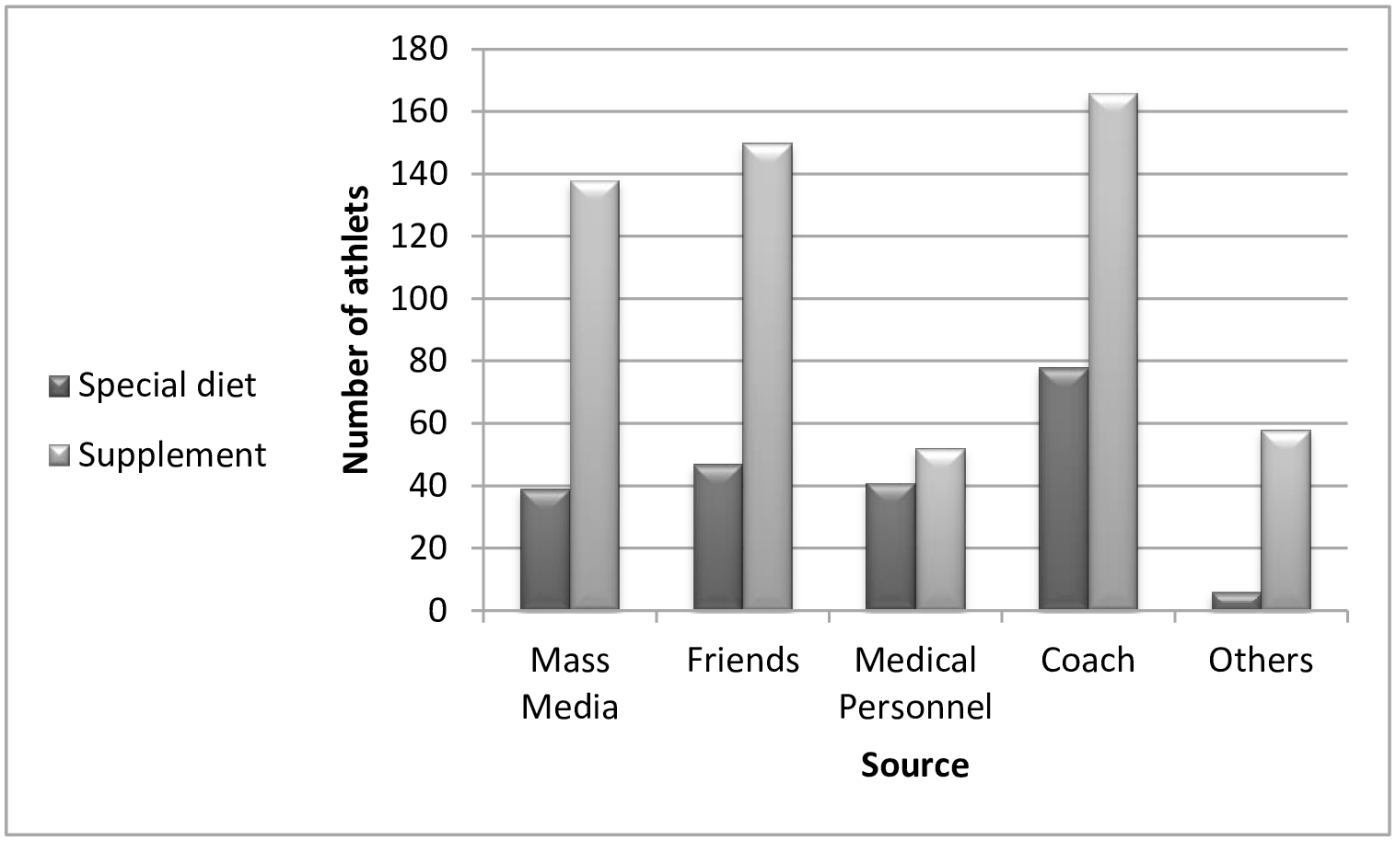
Sources of knowledge on the special diet.

More than 70% of the studied individuals considered themselves knowledgeable about sport nutrition issues Table 3. As shown in “Fig 1”, coaches and athletic trainers were ranked as a major source of nutrition information either for a special diet (n = 78) or sport supplements (n = 166). In total, young athletes could identify more than 16 different classes of sport supplements, with sport drinks (n = 298), creatine (n = 285), vitamins and minerals (n = 186) were the most significantly recognized supplements (p < 0.001).

**Table 3:** Distribution of responses to knowledge and attitude of young athletes to sport nutrition.

	Agree	Disagree	Unsure
	Number (%)	Number (%)	Number(%)
**Can dehydration affect performance?**	224 (62.6)	102 (28.5)	32 (8.9)
**The following is considered as a sport supplement?**			
Sports drinks	298 (83.2)	25 (7)	35 (9.8)
Vitamins and minerals	186 (52)	87 (24.3)	85 (23.7)
Carbohydrates	99 (27.7)	171 (47.8)	88 (24.6)
Proteins	150 (41.9)	97 (27.1)	111 (31)
Cod liver oil	78 (21.8)	215 (60)	65 (18.2)
Herbs	75 (20.9)	179 (50)	104 (29.1)
Thermogenic products	106 (29.6)	147 (41.1)	105 (29.3)
Creatine	285 (79.6)	39 (10.9)	34 (9.5)
Weight loss products	31 (8.7)	251 (70.1)	76 (21.2)
**Before the event**			
Carbohydrates 2–4 hrs	169 (47.2)	98 (27.4)	91 (25.4)
Protein 2–4 hrs	122 (34.1)	173 (48.3)	63 (17.6)
Carbohydrates 1 HR	190 (53.1)	106 (29.6)	62 (17.3)
Multivitamins	176 (49.2)	115 (32.1)	67 (18.7s)
Water	238 (66.5)	82 (22.9)	38 (10.6)
Caffeinated energy drink	147 (41.1)	155 (43.3)	56 (15.6)
Taking supplements	117 (32.7)	157 (43.9)	84 (23.5)
**During the event**			
Protein	109 (30.4)	187 (52.2)	62 (17.3)
Carbohydrates	106 (29.6)	186 (52)	66 (18.4)
Energy drinks	113 (31.6)	190 (53)	55 (15.4)
Soda- containing beverages	49 (13.7)	246 (68.7)	63 (17.6)
Fresh fruit juices	220 (61.5)	91 (25.4)	47 (13.1)
**Post-event**			
Proteins	259 (72.3)	70 (19.6)	29 (8.1)
Carbohydrates	226 (63.1)	81 (22.6)	51 (14.3)
Energy drinks	120 (33.5)	171 (47.8)	67 (18.7)

Approximately, two-thirds of participants agreed that drinking plenty of water prior to the beginning of the event is essential for enhancing athletic performance, while only 28.5% disagreed about the negative effect of dehydration on sporting activity.

More than half (53%) of the athletes significantly believed that the carbohydrate loading one hour before the event would supply sufficient amount of energy (p < 0.001). Also, 61% of participants agreed with the statement that "Do you think consumption of fresh fruit juice helps to improve performance during the event or a basic training workout?”. Protein was significantly selected by more than 70% of the studied athletes as the favorable post-competition meal (p < 0.001) compared to other meals Table 3.

**Fig 2:**
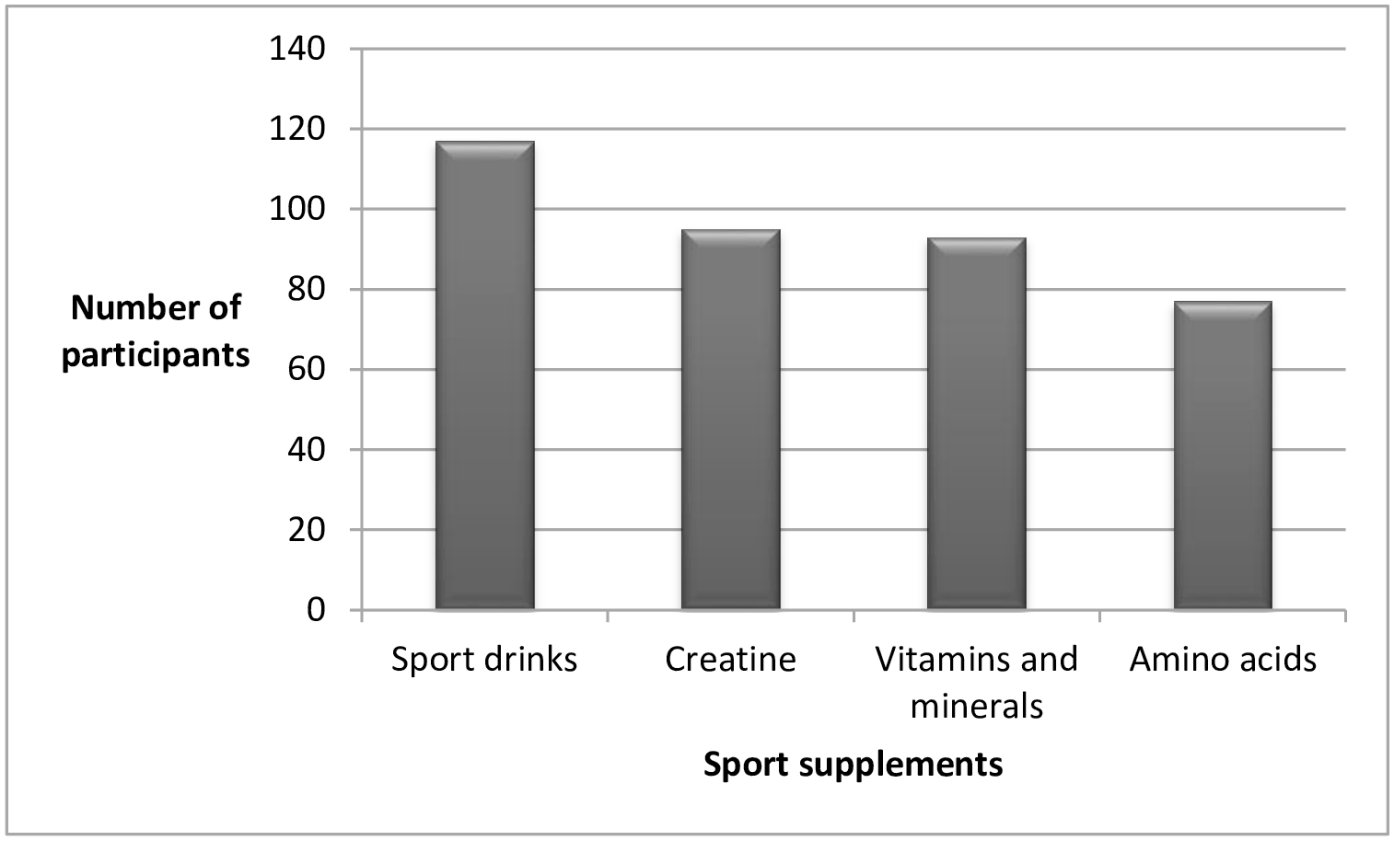
Frequency of sport supplements among study participants.

The attitudes and practice of young athletes to sport nutrition are summarized in Table 4. One-hundred seventy five (48.9%) survey respondents reported taking at least one of these supplements, predominantly sport drinks (66.9%) and creatine (54.3%) “Fig 2”.

**Table 4:** Distribution of responses to attitude and practice of young athletes to sport nutrition.

	Number (n)	%	*P* value
**Do you currently take dietary supplements?**			
Yes	175	48.9	0.601
No	183	51.1	
**If your answers was “No”, the main reasons are? (n = 183)**			
Cost	104	56.8	**<0.001**
Complications	114	62.3	
Unsure	136	74.3	
Others	19	10.4	
**What is the main reason of using dietary supplements? (n = 175)**			
Physical appearance	159	90.9	**<0.001**
Better performance	146	83.4	
Tolerating pain	116	66.3	
Improving concentration	104	59.4	
Peer pressure	94	53.7	
Not harmful	72	41	
**Where do you get the sports supplements? (n = 175)**			
Pharmacy	89	50.9	**<0.001**
Retail store	137	78.3	
Athletic trainer	135	77	
Sport centers	144	82.3	
Supermarkets	87	49.7	
Others	48	27.4	
**How long have you been taking these supplements? (n = 175)**			
Regularly	35	20	**<0.001**
Days	29	16.6	
Weeks	34	19.4	
During competition	77	44	

Maintaining/or obtaining healthy and good physical appearance (n = 159; 44.4%) and achieving better athletic performance (n = 146; 40.8%) were the most common reasons significantly cited for taking sport supplements (p < 0.001). The majority of athletes taking supplements reported sport centers (n = 144), retail stores (n = 137), and athletic trainers (n = 135) as the main sources of obtaining their supplement needs (p < 0.001). In terms of frequency, 44% of respondents used to have supplements only during competition seasons (p < 0.001).

## Discussion

For all athletes, optimum nutrition is the essential variable for promoting athletic success [23]. Based on our results (correct responses), the study participants had a reasonable level of basic nutrition knowledge in almost all domains except for fast food. For example, only 20.5% of young athletes reported breakfast as their missed meal for more than three times a week. Although these results were consistent with previous work from India [14], however, several earlier studies have shown higher levels of skipping meals [24,25]. This difference could be attributed to cultural norms and values of old-eating pattern in Egypt that allow family gathering at least once daily [26]. Also, the abundance of information on the internet, social media and other sources had contributed to increased health awareness among adolescents. Yet, they still need close supervision.

In contrast to other studies [27,28], vegetables and fruits have represented a crucial part for participants with snacking habits. We identified fast food (known to be energy dense, high in saturated fat and have low micronutrient content) [29] as the most common unhealthy dietary pattern, frequently practiced by study individuals and constituted the best choice for outdoor meals. These results highlighted the influence of modern life style that made adolescents the primary consumer of fast-food.

The results of this study confirmed previous reports [30,31] that coaches play a fundamental role in improving physical, mental and ethical standards of young athletes [32]. In our study, the majority of dietary information passed on to young athletes was obtained from coaches and friends. These findings emphasize the necessity of implementing national coach education programs on a regular basis.

In terms of scores, sport nutrition knowledge, beliefs and attitude had the lowest number of correct responses, showing continuity with previous research [33–35]. Approximately, half of the study participants incorrectly agreed that pre-event consumption of multivitamins will enhance athletic performance, a result that is 15–20% higher than findings of similar studies [36]. Moreover, only 52.2% of athletes had acknowledged the energetic effects of carbohydrates as a pre-workout meal, a response that is lower than those from other surveys [37,38].

Although, many literatures [37,39] clarified the role of proteins as a major source for muscle growth and not for physical activity, however, a majority of athletes consider proteins as an energy-dense meals, a finding replicated in our study. It is obvious that issues related to micro-and macro-nutrients represent an area of confusion for young athletes. Thus, an in-depth sport-based nutrition education is required to address theses topics and improve dietary skills.

In recent years, consumption of ergogenic aids (e.g. Ephedra alkaloids, anabolic steroids, protein hormone) has been increased to enhance athletic performance. Young athletes, especially elite and competitive, are the main target market for such supplements [40]. In this context, it should be pointed out that the safety of some ergogenic aids in children and young adolescents, for example; creatine supplementation is not established and is therefore, not recommended by many authors. Furthermore, it was speculated that creatine supplementation may aggravate the side effects of performance enhancing products such as anabolic steroids in the population under the age of 18 [41,42].

Two-thirds of our study participants have recognized different types of ergogenic aids, however, only caffeine-supplement consumption had been agreed by 40% of the athletes. A previous German study conducted on 1265 adolescents found that 53% had tried caffeinated drinks once, whereas 23% and 3% used to drink one can and seven cans per week, respectively [43]. Typically, energy drinks, especially those which have high levels of caffeine, may put some children at risk of serious adverse health effects [44].

Similar to other studies, [33,40] a majority of participants were more adherent to sports hydration guidelines for pre-, during, and post-event as recommended by The American College of Sports Medicine [45]. Although, sport drinks were the most supplement frequently consumed, however the rate of consumption was lower than expected in our study. These results were in agreement with other surveys [46]. Such findings raise concerns about how far the knowledge of young athletes about the necessity of proper hydration and the importance of sport drinks containing sodium and carbohydrates in a special situations such as events lasting more than one hour [3].

The overall prevalence rate of supplement use in our survey was found to be much lower than some studies [41] and greater than others [31]. This difference may be explained by variable sample sizes involved in other studies. Type of the products may be another explanation; as a large proportion of supplements consumed were multivitamins and minerals, which found to be the most widespread used supplements among athletes. Also, some products such as energy drinks are not necessarily sport-specific supplements [47,48].

We also found that consumption of supplements was influenced by competition in the majority of cases, while a small number of athletes (9.8%) reported taking supplements on a regular basis.

In contrast to several previous researches, [41,48,49] concerning with body shape and improvement of physical performance were the most frequent reasons addressed for sport supplement use. Variety of sources providing information and inclusion of different types of sports, especially weight class might have influenced these findings.

The majority of the study participants who were not interested in taking supplements have been not confident about the benefits of supplementations. Overall, such behavior warrants proper investigations of the real athlete's priorities, careful monitoring and counseling of young athletes to avoid misuse of supplements and ensure that dietary extremes would not be attained.

To the best of our knowledge, this is the first study that identified perspectives of nutrition and sport supplements use in young sport communities in Egypt. Nonetheless, important study limitations should be addressed. One of the study restrictions was its relatively small sample size which may be not sufficient to interpret the real extent of the level of dietary knowledge, behavior and attitude among adolescent athletes. Moreover, all study participants were allocated from one governorate (Greater Cairo), which may limit the generalisability of the results. However, the study population was selected to be representative of the majority of young Egyptian athletes; as approximately 25% of the Egyptian population resides in Cairo [50]. Responses to some questions may be biased if respondent didn’t answer honestly or had preferences for nutrition issues. Therefore, all questionnaires were attended by the researchers with face-to-face structured interview decreasing the potential for bias. This study was designed as a quantitative survey to outline the dietary profile and supplementation among young athletes. Thus, we were limited in our ability to assess the influence of certain factors such as gender, age category, level of education, type of sport and the number of training hours on athlete's nutrition. It is also widely accepted that BMI doesn’t take into account body fat, which is used to determine the body composition of the adolescent athletic population [51]. Consequently, we didn’t use BMI as an indicator for obesity in our study participants Finally, despite the study instrument was anonymous, however, illegal sport supplementation couldn’t be assessed.

## Conclusion

To conclude, majority of Egyptian young athletes have at least one fixed meal, adequate snacking habits, and adherent to hydration guidelines. Like other countries, adolescent athletes respond frequently to fast food offers. Although, sport drinks, vitamins and minerals were the most prevalent supplements, however, the rate of their intake was insufficient. Pattern of basic nutrition of young athletes was quite satisfactory, while, sport nutrition skills and knowledge were below average. A lot of work and attention are required to provide functional nutrition knowledge with health-promoting decisions and behaviors among young athletes. For example, an adequate design and implementation of a comprehensive sport nutrition education program involving young athletes, parents, coaching staff, health trainers and other team officials.
